# Proportion of Deaths in Child-Bed to Births

**Published:** 1811-03

**Authors:** 


*To the Editors of the Medical and Physical Journal.*

GENTLEMEN,

IF you think the inclosed Tables, collected with some labour for my own use, likely to afford either amusement or information to your readers, they are very much at your service. In return I should be happy to receive from any of your intelligent correspondents, replies to the annexed queries. I remain, &c.

*Feb.* 11, 1811. OBSTETRICUS.

A TABLE

A TABLE taken from the Annual Bills of Mortality, shewing from averages of ten years from 1661, the proportion which the deaths in child-bed bear to the births.

| | | | |
|---|---|---|---|
| 1st Series of Ten Years, ending | 1670 | . 1 in | 39 |
| 2d Do . . . | 1680 | . 1 in | 49 |
| 3d Do . . . | 1690 | . 1 in | 47 |
| 4th Do . . . | 1700 | . 1 in | 65 |
| 5th Do . . . | 1710 | . 1 in | 67 |
| 6th Do . . . | 1720 | . 1 in | 72 |
| 7th Do . . . | 1730 | . 1 in | 73 |
| 8th Do . . . | 1740 | . 1 in | 70 |
| 9th Do . . . | 1750 | . 1 in | 74 |
| 10th Do . . . | 1760 | . 1 in | 81 |
| 11th Do . . . | 1770 | . 1 in | 72 |
| 12th Do . . . | 1780 | . 1 in | 92 |
| 13th Do . . . | 1790 | . 1 in | 107 |
| 14th Do . . . | 1800 | . 1 in | 113 |
| 15th Do . . . | 1810 | . 1 in | 106 |

---

A TABLE of two years in each of the above periods of ten years, in which, the greatest number of deaths in child-bed took place, with the number of those who actually died.

| | | | |
|---|---|---|---|
| 1665 | 625 Deaths. | 1741 | 256 Deaths. |
| 1670 | 288 | 1750 | 228 |
| 1679 | 300 | 1754 | 213 |
| 1680 | 302 | 1760 | 238 |
| 1681 | 354 | 1761 | 289 |
| 1688 | 355 | 1762 | 272 |
| 1694 | 262 | 1777 | 222 |
| 1697 | 293 | 1779 | 209 |
| 1704 | 266 | 1781 | 209 |
| 1705 | 287 | 1787 | 213 |
| 1714 | 309 | 1792 | 201 |
| 1719 | 292 | 1796 | 202 |
| 1721 | 299 | 1803 | 250 |
| 1722 | 293 | 1806 | 235 |
| 1733 | 292 | | |
| 1737 | 284 | | |

A TABLE

A Table of two years in each of the above periods of ten years, in which the mortality in child-bed was least, with the number of those who actually died.

| | | | |
|---|---|---|---|
| 1662 | 175 Deaths. | 1743 | 176 Deaths. |
| 1663 | 206 | 1744 | 184 |
| 1673 | 224 | 1752 | 156 |
| 1674 | 217 | 1753 | 169 |
| 1686 | 288 | 1767 | 174 |
| 1690 | 236 | 1769 | 185 |
| 1692 | 218 | 1771 | 172 |
| 1698 | 169 | 1778 | 175 |
| 1709 | 215 | 1782 | 140 |
| 1710 | 217 | 1784 | 133 |
| 1703 | 217 | 1795 | 142 |
| 1711 | 195 | 1799 | 131 |
| 1713 | 178 | 1807 | 164 |
| 1727 | 221 | 1809 | 123 |
| 1728 | 216 | | |
| 1735 | 192 | | |
| 1736 | 202 | | |

The deaths in miscarriages, by which must be meant abortions under four months of gestation, have only been recorded since the year 1723: they amount upon an average to three annually: but there seem to be no accurate means of knowing how many miscarriages happen within a year. Information upon this subject would be very useful.

The years 1803, 4, 5, and 6, were remarkable for an increased mortality in child-bed; the deaths for these four years amounted to 937, which was on an average 1 in 91.

To what causes was this increase owing?

During the last four years, viz. 1807, 8, 9, and 10, the mortality has been much less, it amounted only to 642, which is on an average 1 in 126.

What are the principal diseases which prove fatal in child-bed? Are they peculiar to that state? What proportion do the deaths in childbed, from diseases peculiar to that state, bear to the deaths, from diseases in childbed not peculiar to that state.

